# Power Line Communication with Robust Timing and Carrier Recovery against Narrowband Interference for Smart Grid

**DOI:** 10.3390/s22114013

**Published:** 2022-05-25

**Authors:** Sicong Liu, Fang Yang, Dejian Li, Ruilong Yao, Jian Song

**Affiliations:** 1 Department of Information and Communication Engineering, School of Informatics, Xiamen University, Xiamen 361005, China; liusc@xmu.edu.cn; 2 Department of Electronic Engineering, Tsinghua University, Beijing 100084, China; fangyang@tsinghua.edu.cn; 3 Beijing National Research Center for Information Science and Technology (BNRist), Beijing 100084, China; 4 Beijing Smart-Chip Microelectronics Technology Co., Ltd., Beijing 102200, China; lidejian@sgitg.sgcc.com.cn (D.L.); yaoruilong@sgitg.sgcc.com.cn (R.Y.)

**Keywords:** power line communication, narrowband interference, preamble, synchronization, smart grid

## Abstract

Power line communication (PLC) is an important interconnection technology for the smart grid, but the robustness of PLC transmission is faced with a great challenge due to strong non-Gaussian noise and interference. In this paper, a narrowband interference (NBI) resistant preamble is designed, and an effective timing and frequency synchronization method is proposed for OFDM-based PLC systems in the smart grid, which is capable of simultaneously conveying some bits of transmission parameter signaling (TPS) as well. In the time domain, the cyclic extension of the training OFDM symbol is scrambled, which makes it feasible to combat against NBI contamination. More accurate timing detection and sharper correlation peak can be implemented under the power line channel and the AWGN channel in the presence of NBI, compared with the conventional Schmidl’s and Minn’s methods with the same preamble length. Furthermore, the TPS transmitted using the proposed method is also immune from the NBI. The proposed method is capable of improving the synchronization performance of the PLC transmission significantly, which is verified by theoretical analysis and computer simulations.

## 1. Introduction

Power line communication (PLC) has been widely applied in applications, such as the smart grid, smart home access networks, and internet-of-things, etc. Orthogonal frequency division multiplexing (OFDM) is a widely applied physical layer modulation technique, because of the good performance against frequency selectivity as well as simple implementation and high transmission efficiency. Many different communication systems have adopted OFDM, including wireless local area network (WLAN) [1], visible light communication systems [2,3], PLC systems specified by the ITU G.9960 standard [4], and digital video broadcasting (DVB) [5,6], etc. Nevertheless, there exist various kinds of noises in the transmission channels of the OFDM-based PLC systems, which makes it more difficult to guarantee the communication performance in the PLC channel [7,8]. The channel environment are filled with attenuation, noise and interference, especially, as investigated in this paper, the narrowband interference (NBI) prevailing in the PLC transmission environment [7,8,9,10], which brings great challenge to the performance of OFDM-based PLC systems, including synchronization, demodulation and decoding. Although the conventional OFDM-based PLC system is simpler to be implemented and adopted by the existing PLC standards, the more recently emerging other versions of OFDM systems, which are more effective, can be introduced to further improve the performance of PLC systems [11,12,13,14].

In OFDM systems, first of all, as a major concern, accurate synchronization is the prerequisite of a stable and effective communication system [15,16,17,18]. In OFDM systems, there have been some methods that are aimed at preamble design and synchronization. The cyclic prefix (CP) is utilized in [15] to implement the classical sliding auto-correlation (SAC) method. A preamble design structure with two identical sequences, which was applied in WLAN IEEE 802.11g [1] and WMAN IEEE 802.16e [19], was proposed by Schmidl [16]. However, a broad plateau around the correct timing position would be generated in the result of the SAC using Schmidl’s design, so it is difficult to estimate the accurate timing position. To solve this problem, a preamble structure as denoted by [AA−A−A] was proposed by Minn et al. to cut down the plateau and sharpen the peak of the SAC results [17]. However, Minn’s design would cause several sub-peaks in the SAC result, which makes it highly probable to obtain false detection when the signal-to-noise ratio (SNR) is small.

Moreover, commonly, there exists prevailing intensive NBI, contaminating the channel of OFDM systems, having serious impacts on the system stability and effectiveness [20]. Unfortunately, the state-of-the-art methods of synchronization could be seriously impacted by the NBI [21,22]. The existing synchronization methods are neither practical nor effective as far as the NBI contamination is concerned. For instance, the impacts of the NBI on the performance of the existing synchronization methods in practical PLC channels are shown in [23]. Hence, in this context, proposing an effective preamble design as well as the synchronization method for OFDM systems impacted by NBI is in desperate need, which is one of the major contributions of this paper.

In addition, different symbol constellation types and channel coding rates should be supported in OFDM systems so as to satisfy different requirements of quality of service (QoS) [24]. Exploiting the transmission parameter signaling (TPS) is a common and effective approach to facilitate the subsequent channel estimation [25], and crucial in correctly demodulating the transmitted data for the receiver. The TPS is transmitted separately following the preamble in the existing commercialized systems, such as IEEE 802.11g and IEEE 802.16e. After finishing the timing and frequency synchronization processes, then the signaling part could be handled and decoded. However, the TPS is dependent on the accurate timing synchronization, which seriously suffers from the impacts of the NBI, making the decoding of TPS inaccurate. Besides, this process is spectrum inefficient since a dedicated resource is required to transmit the TPS part following the preamble. If, as designed in this paper, the TPS is integrated into the sub-carriers of the preamble, the process of the TPS acquisition can be independent of the timing synchronization and meanwhile the NBI is mitigated. Moreover, the spectral efficiency is greatly improved because the preamble has already carried the TPS, so no extra dedicated resource is required for TPS, which is another contribution of this paper.

Therefore, the main contributions of this paper are two-fold as follows:

- An improved OFDM-based preamble structure that inherits the advantages of both Schmidl’s and Minn’s methods is proposed for robust PLC transmission. More importantly, a novel scrambling operation is applied in the training sequence of the designed preamble so that the influence of the NBI is eliminated.
- In the frequency domain, to achieve diversity gain under the frequency-selective fading channels, two identical training sequences (TS) are distributed alternately in the active sub-carriers. In order to indicate several bits of signaling information for the receiver to acquire the basic transmission parameters quickly, the relative distance between the two TSs could vary.

The remainder of this paper is outlined as follows. The OFDM signal model in PLC transmission is briefly introduced and the two most popular conventional preamble designs are reviewed in Section 2. The proposed preamble design and the corresponding preamble detection and synchronization algorithm are presented in Section 3 and Section 4, respectively. In Section 5, the performance of the conventional and proposed preambles, as well as the synchronization methods, are tested through computer simulations, which is followed by the conclusions in Section 6.

## 2. Signal Model and Related Work

The modulated symbols ${\left\{X_{k}\right\}}_{k=0}^{N-1}$ at *N* sub-carriers are processed with an *N*-point inverse fast Fourier transform (IFFT) to produce the OFDM symbol as given by

(1) $$x_{n}=\frac{1}{\sqrt{N}}\sum_{k=0}^{N-1}X_{k}\cdot e^{j\frac{2\pi }{N}nk}.$$

Taking the additive white Gaussian noise (AWGN), frequency-selective multi-path channel, the carrier frequency offset (CFO) and the NBI into consideration, the received signal can be represented as,

(2) $$r_{n}=\sum_{l=0}^{L_{h}-1}h_{l}\cdot x_{n-n_{0}-l}\cdot e^{j2\pi nf_{c}}+I_{0}\cdot e^{j2\pi nf_{\mathrm{NB}}}+\nu_{n},$$

where $n_{0}$, $f_{c}$ and $\nu_{n}$ are the unknown time of symbol arrival (which is the desired correct timing position to be estimated by the receiver), CFO and AWGN, respectively [21]. The multi-path channel impulse response (CIR) is denoted by ${\left\{h_{l}\right\}}_{l=0}^{L_{h}-1}$, which is modeled by $L_{h}$ delay taps. The NBI amplitude at the frequency point $f_{\mathrm{NB}}$ is denoted by $I_{0}$. Normally, the CFO is normalized by the sub-carrier spacing 1/N, i.e., $f_{c}=k_{0}/N+f_{\mathrm{frc}}$, where $k_{0}$ is an integer representing the integer part of the CFO, and the remaining fractional part of the CFO is denoted by $f_{\mathrm{frc}}$.

**Figure 1:**
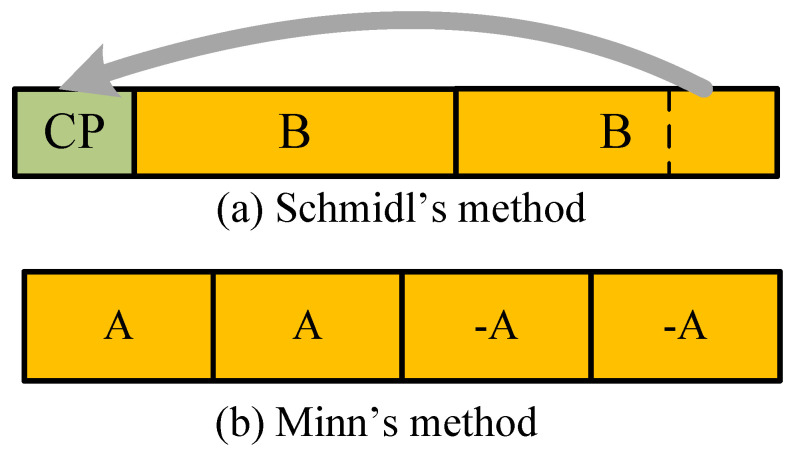
Conventional state-of-the-art designs of the OFDM preamble for synchronization.

The target of the synchronization process at the receiver is estimating the correct timing position and determining the CFO without knowledge of prior channel state information. There have been some conventional methods proposed by T. M. Schmidl et al. [16] and H. Minn et al. [17]. Specifically, to achieve this goal, as illustrated in Figure 1a, Schmidl proposed a preamble design whose time-domain structure consists of the two same components [16]. The two cyclic parts ‘**B**’ are used to calculate the SAC result for synchronization. However, because of the CP part, a wide plateau around the correct timing position would be generated in the SAC result, which would lead to ambiguity and inaccuracy in estimating the timing position. In order to cut down the plateau, a novel time–domain preamble as denoted by [AA−A−A] was proposed by Minn, which is depicted in Figure 1b [17]. This deliberate design with two opposite components is capable of sharpening the SAC peak with respect to that of Schmidl’s. Nevertheless, some sub-peaks are inevitably generated in the SAC result, leading to an increase in the probability of false detection, especially in the case of small SNR.

Taking the NBI at the frequency point $f_{\mathrm{NB}}$ into consideration, the SAC result of the conventional preamble based on two identical parts at the desired timing position $n_{0}^{{}^{\prime }}$ is given as (we first omit the noise and multipath channel terms for simplicity, and then the analysis can be easily extended to multi-path channels as described in Appendix A),

(3) $$\begin{array}{c} R_{c,n_{0}^{{}^{\prime }}}=\sum_{l=0}^{L_{c}-1}r_{n_{0}^{{}^{\prime }}+l}\cdot r_{n_{0}^{{}^{\prime }}+l+N_{c}}^{*}\\ \;\;\;\;\;=\sum_{l=0}^{L_{c}-1}\left(x_{l}+I_{0}e^{j2\pi f_{\mathrm{NB}}l}\right)\;\cdot {\left(x_{l}+I_{0}e^{j2\pi f_{\mathrm{NB}}\left(l+N_{c}\right)}\right)}^{*}\;\;\\ \;\;\;\;\;=\sum_{l=0}^{L_{c}-1}\left({\left|x_{l}\right|}^{2}+I_{0}x_{l}e^{-j2\pi f_{\mathrm{NB}}\left(l+N_{c}\right)}\right.\\ \;\;\;\;\;\;\;\;\;\;\;\left.\;\;\;\;\;\;\;\;+I_{0}x_{l}^{*}e^{j2\pi f_{\mathrm{NB}}l}+I_{0}^{2}e^{-j2\pi f_{\mathrm{NB}}N_{c}}\right)\\ \;\;\;\;\;\approx \sum_{l=0}^{L_{c}-1}{\left|x_{l}\right|}^{2}+L_{c}\cdot I_{0}^{2}e^{-j2\pi f_{\mathrm{NB}}N_{c}} \end{array},$$

where $N_{c}$ and $L_{c}$ denote the length of the correlation lag and the identical components, and ${\left(\cdot \right)}^{*}$ represents the complex conjugation operation. Since the transmitted preamble $\left\{x_{l}\right\}$ and the NBI $\left\{e^{j2\pi f_{\mathrm{NB}}l}\right\}$ are non-coherent signals, the cross terms in (3) are eliminated after sum averaging and can be neglected with respect to the preamble SAC component ${\left|x_{l}\right|}^{2}$ and the significant NBI component $L_{c}I_{0}^{2}e^{-j2\pi f_{\mathrm{NB}}N_{c}}$. It is observed from (3) that the correlation peak might be deteriorated by the unpredictable strong NBI signal, and hence cause degradation to the detection performance. In order to solve the problems of the conventional methods, the preamble is specifically designed and optimized in this paper, along with the proposed synchronization method to improve the synchronization performance against the NBI impacts, as described in detail in the following.

## 3. Proposed Design of Preamble for Synchronization

**Figure 2:**
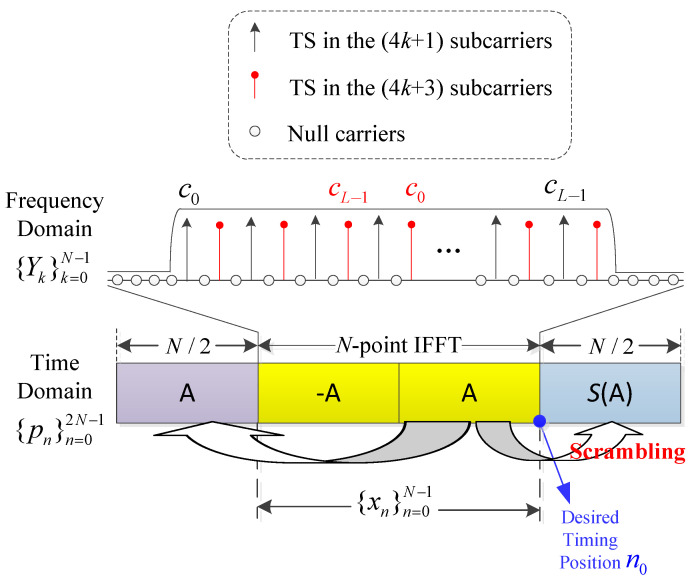
Proposed OFDM preamble design.

As depicted in Figure 2, the proposed preamble structure consists of an OFDM symbol of length *N* as well as the two cyclic extensions of this symbol. Two groups of TSs are alternatively allocated in sub-carriers with indices of {4k+1} and {4k+3}, respectively, in the frequency domain. The first group of TSs allocated in the {4k+1} sub-carriers starts from the initial position, while the other group of TSs allocated in the {4k+3} sub-carriers are cyclically right shifted by ΔL, thus formulating the frequency-domain sequence ${\left\{Y_{k}\right\}}_{k=0}^{N-1}$ as given by

(4) $$\left\{\begin{array}{c} Y_{4k+1}=c_{k},\;\;\;\;\;\;\;\;\;\;\;\;\;\;\;\;\;\;\;k=0,1,\cdots ,L-1\\ Y_{4k+3}=c_{(k-\Delta L)\;\mathrm{mod}\;L},\;k=0,1,\cdots ,L-1\\ Y_{k}\;\;\;\;=0,\;\;\;\;\;\;\;\;\;\;\;\;\;\;\;\;\;\;\;\;\;\;\mathrm{others} \end{array}\right.\;,$$

where ${\left\{c_{k}\right\}}_{k=0}^{L-1}$ is the length-*L* (*L* < N/4) pseudo-random TS with a good property of auto-correlation, and mod represents the modular operation. Different signaling information can be conveyed through the variation of the cyclically shifted length ΔL. To convey $\log_{2}L$ bits of signaling, there can be totally *L* choices for the shifted length. Note that all the preamble designs and the corresponding synchronization methods, including the proposed and the conventional ones, are considered to have the same preamble length for fair comparison.

Afterwards, the active sub-carriers in (4) are differentially encoded and then processed by the *N*-point IFFT operation defined in (1) to obtain the time–domain preamble. As shown in Figure 2, the derived time–domain OFDM symbol can be regarded as two opposite parts, as represented by ‘−**A**’ and ‘**A**’, since only odd sub-carriers are occupied. Then, the latter half part ‘**A**’ is multiplied by a scrambling sequence ${\left(-1\right)}^{n}$ and appended to the rear to form the ‘**S(A)**’ part. Meanwhile, the latter half part ‘**A**’ itself is copied to the front to play the role of CP as well. The whole time–domain transmitted preamble signal is hence denoted by ${\left\{p_{n}\right\}}_{n=0}^{2N-1}$ given by

(5) pn=xn+NN22,0≤n<NN22xn−NN22,NN22≤n<3N3N22(−1)n·xn−N,3N3N22≤n<2N,

where ${\left\{x_{n}\right\}}_{n=0}^{N-1}$ denotes the time–domain length *N* OFDM symbol generated by the *N*-point IFFT operation, as illustrated by the yellow part in Figure 2. The operation of scrambling could effectively relieve the contamination of the NBI, as explained in detail in the following section.

## 4. Timing and Carrier Frequency Synchronization through Preamble Detection

Both timing and frequency synchronization along with the algorithm of signaling detection are described in detail in this section to showcase the advantages of the proposed preamble design and synchronization method with respect to the conventional methods.

### 4.1. Timing and Fractional CFO Estimation

The cyclic property of the preamble can be exploited in the algorithm of timing at the receiver. Specifically, the following three pairs of cyclic parts are exploited for the calculation of SACs,

(6) R1,n=∑l=0NN22−1(−1)(n+l)rn+l*·rn+l−NN22,

(7) R2,n=∑l=0NN22−1(−1)(n+l)rn+l*·rn+l−N,

(8) R3,n=∑l=0NN22−1(−1)(n+l)rn+l*·rn+l−3N3N22.

As shown in Figure 2, the de-scrambling operations between the three front parts ‘**A**’, ‘−**A**’, ‘**A**’ and the last part ‘**S(A)**’ are implemented through multiplying by the descrambling coefficient ${\left(-1\right)}^{n}$ in the summation of (6)–(8). After that, in order to further sharpen the correlation peak, these three SAC results are multiplied together to generate the final positive peak as given by

(9) $$R_{c,n}=-R_{1,n}^{*}\cdot R_{2,n}\cdot R_{3,n}.$$

**Figure 3:**
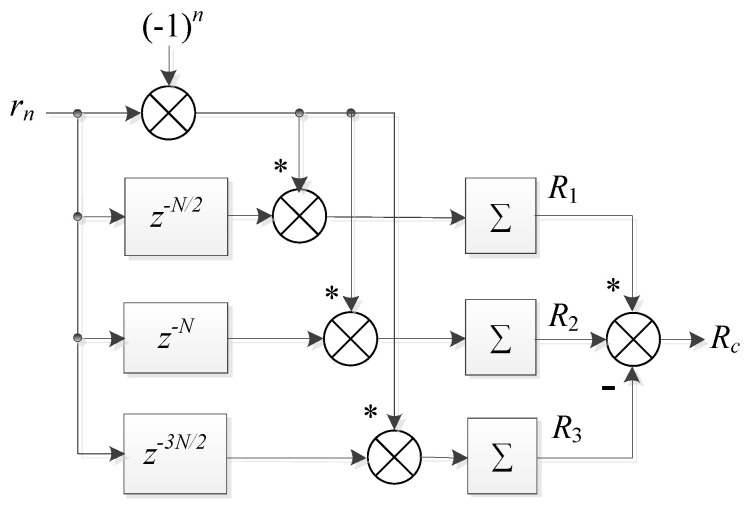
Proposed time–domain sliding auto-correlation module for timing and carrier frequency synchronization.

The proposed SAC block diagram for timing and carrier frequency synchronization is depicted in Figure 3. From the diagram and the above SAC calculation method (6)–(9), it can be found that the theoretical desired timing position $n_{0}$ is at the first sample of the part ‘**S(A)**’, as illustrated in Figure 2. Before calculating the correlation, a descrambling operation, i.e., multiplying by the descrambling coefficient ${\left(-1\right)}^{n}$, is prior implemented toward the received preamble, which is different from existing methods. It can be noticed that with the same preamble length, the computational complexity of the proposed method, mainly reflected by the number of additions and multiplications in calculating correlations, is about 50% more than that of Minn’s method. This is because three groups of SAC are required to be calculated in the proposed method, whereas only two groups are calculated in Minn’s method. However, the complexity of the proposed algorithm is still low, which is analyzed in detail in Section 4.3 since the summations in (6)–(8) could be implemented with recursive method [16].

At the receiver, the estimated timing position ${\hat{n}}_{0}$ in the preamble can be estimated from the correlation peak of $R_{c,n}$, and the fractional CFO can be calculated by the phase of the correlation peak as well,

(10) $${\hat{n}}_{0}=\underset{n}{\arg\max}\left\{\left|R_{c,n}\right|\right\},$$

(11) $${\hat{f}}_{\mathrm{frc}}={\left.\frac{\arg\left(R_{c,n}\right)}{2\pi N}\right|}_{n={\hat{n}}_{0}},$$

where $\arg\left(\cdot \right)$ is the phase calculation operation for a complex number, and $\arg\max\left\{\cdot \right\}$ derives the variable that maximizes the interior objective function.

**Figure 4:**
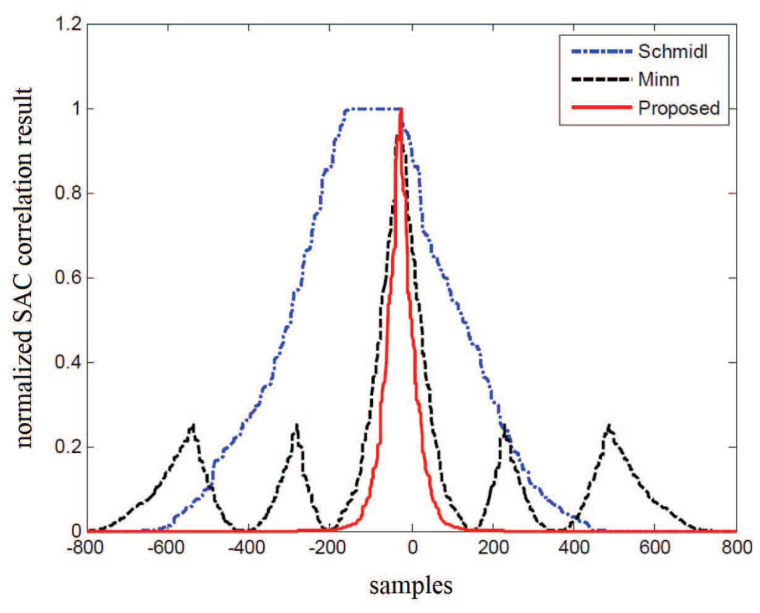
Comparison for timing peaks of three methods.

To demonstrate the advantage of the proposed method in SAC peak performance over Schmidl and Minn’s methods, as illustrated in Figure 4, the simulation results are presented, where the channel noise is absent. It is noted from Figure 4 that a broad plateau whose length is the same with that of the CP is generated for Schmidl’s method. Meanwhile, Minn’s method produces four sub-peaks, with each having approximately 1/4 the amplitude of the main peak. Therefore, it can be observed that both the plateau and the sub-peaks are avoided in the proposed method, and a much sharper main peak is derived compared with that of Minn’s method.

Now we look at the capability of the proposed method against the NBI impact. Let us consider an NBI with the frequency $f_{\mathrm{NB}}$, and without loss of generality, we investigate the first SAC result $R_{1,n}$ in (6) at the desired correct timing position $n_{0}$, which is applied by a descrambling operation,

(12) R1,n0=∑l=0NN22−1(−1)l(−1)lxl+I0ej2πfNBl*·xl+I0ej2πfNB(l−NN22)=∑l=0NN22−1xl2+I0xl*ej2πfNB(l−NN22)+(−1)lI0xle−j2πfNBl+(−1)lI02e−j2πfNBNN22≈∑l=0NN22−1xl2.

where the cross terms are eliminated after de-scrambling and sum averaging since the NBI and the preamble are non-coherent signals. From (12), importantly, it is observed that the NBI component ${\left(-1\right)}^{l}I_{0}^{2}e^{-j2\pi f_{\mathrm{NB}}N/2}$ is eliminated after sum averaging due to the scrambling operation. Therefore, it is observed that the SAC result of $R_{1,n}$ reaches its maximum peak value at the desired timing position $n_{0}$. Similarly, the SAC branches $R_{2,n}$ and $R_{3,n}$ in (7) and (8) at the desired correct timing position $n_{0}$ are respectively given as

(13) R2,n0≈−∑l=0NN22−1xl2,

(14) R3,n0≈∑l=0NN22−1xl2.

Hence, $R_{2,n}$ and $R_{3,n}$ also reach their maximum peak value at the desired timing position $n_{0}$. Finally, the accumulated SAC correlation $R_{c,n}$ as given by (9) is free from the NBI contamination and will also reach the maximum peak value at the desired timing position $n_{0}$, so the detected timing position ${\hat{n}}_{0}$ estimated by (10) is accurately the desired timing position $n_{0}$. Based on this analysis, the capability and robustness of the proposed timing method against the NBI is validated.

Furthermore, the proposed synchronization method can be easily extended to multi-path channels and the NBI resistant mechanism as derived in (12) and (3) still effectively holds, which is proved in the Appendix A.

### 4.2. Integer CFO Estimation and Signaling Detection

The integer CFO can be estimated and the signaling conveyed through the frequency domain preamble can be detected when the receiver finishes the timing synchronization. The received OFDM symbol is first compensated by the estimated fractional CFO $f_{\mathrm{frc}}$, and then transformed to the frequency domain through *N*-point FFT, which yields the active carriers at the receiver given by

(15) $${\hat{Y}}_{k}^{\left(d\right)}=Y_{k-k_{0}}^{\left(d\right)}H_{k-k_{0}}e^{-j\frac{2\pi }{N}\Delta n\cdot k}+N\cdot I_{0}\cdot \delta_{k-k_{\mathrm{NB}}}+V_{k},$$

where $Y_{k}^{\left(d\right)}$, $H_{k}$, and Δn denote the transmitted active carrier after differential encoding operation, the channel frequency response, and the timing error which causes a phase rotation of the active carriers, respectively. $\delta_{k}$ and $k_{\mathrm{NB}}$ denote the Kronecker delta function and the sub-carrier index at the frequency point $f_{\mathrm{NB}}$, respectively. The frequency domain noise term is denoted by $V_{k}$. It is noted from (15) that a shift of all carriers would be caused by the integer part of CFO $k_{0}$.

The NBI’s impact on determining the integer CFO and signaling can be eliminated as well. We only need to exclude the NBI contaminated sub-carrier through nulling the sub-carriers with excessively large power to zero. Afterward, the received active carries are applied by a differential decoding operation,

(16) $$\begin{array}{c} Z_{k}=\;{\hat{Y}}_{k}^{\left(d\right)}\cdot {\hat{Y}}_{k-2}^{(d)*}\\ \;\;\;\;\;=H_{k-k_{0}}H_{k-k_{0}-2}^{*}\cdot Y_{k-k_{0}}e^{j\frac{2\pi }{N}2\Delta n}+{\tilde{V}}_{k}\\ \;\;\;\;\;\approx {\left|H_{k-k_{0}}\right|}^{2}Y_{k-k_{0}}e^{j\frac{2\pi }{N}2\Delta n}+{\tilde{V}}_{k} \end{array},$$

where ${\tilde{V}}_{k}$ denotes the remaining noise. The approximation in (16) holds as long as the channel frequency response at the adjacent sub-carriers are closely similar to each other, which is practical for the channel whose frequency selectively is not too severe between adjacent sub-carriers.

Comparing (16) with (15), one can observe that the phase rotation term $e^{-j\frac{2\pi }{N}\Delta n\cdot k}$ associated with the sub-carrier index *k* caused by the timing error Δn is eliminated through the receiver-side differential decoding, and only a fixed phase offset that has no influence on the detection of integer CFO and signaling, is left, making the proposed method immune to timing errors.

Through the cross correlation between the local TS and the received differentially decoded sub-carriers, the signaling information and the integer CFO could be simultaneously acquired, i.e.,

(17) $$R_{d,k}=\frac{\sum_{l=0}^{L-1}Z_{\left(k+4l\right)\;\mathrm{mod}\;4L}\cdot c_{l}^{*}}{\sum_{l=0}^{L-1}{\left|{\hat{Y}}_{\left(k+4l\right)\;\mathrm{mod}\;4L}\right|}^{2}},\;\;0\leq k<N.$$

**Figure 5:**
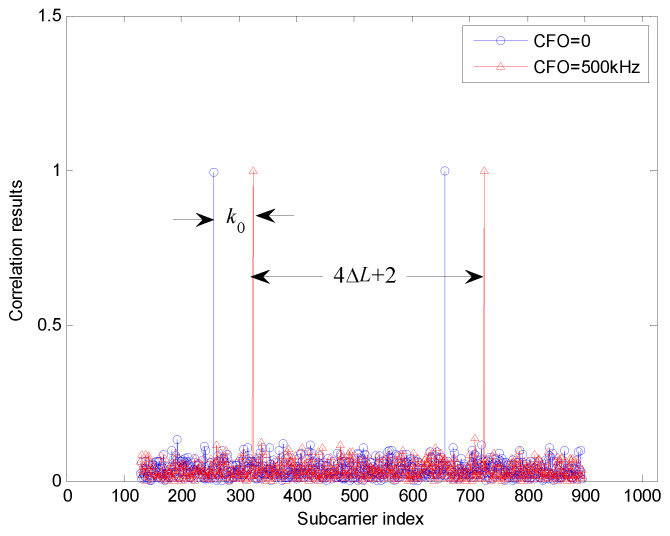
Frequency–domain correlation results with signaling and different integer CFOs.

Theoretically, as shown in Figure 5, two pairs of peaks are expected to be generated by the correlation in (17) due to the integer CFO and the cyclically shifting between the two groups of TSs allocated at the {4k+1} and {4k+3} sub-carriers. Hence, the integer CFO $k_{0}$ could be estimated through the distance between the first peak and its reference position when CFO = 0. And meanwhile, the signaling parameter ΔL could be acquired from the shifting distance between the two corresponding pairs of peaks, which is (4ΔL+2) as shown in Figure 5.

### 4.3. Computational Complexity Analysis

Let us consider the computational complexity of the proposed synchronization method and compare it with that of the conventional Minn’s method. Note that the comparison is conducted under the same preamble length for both methods, i.e., 2N according to Section 3. For both methods, the main consumption of computational complexity goes to the part of the calculation of SAC. Usually, the computational complexity can be evaluated by the quantity of additions and multiplications, given in detail as follows.

For the conventional Minn’s method [17], there are two SAC windows for the positive preamble part and negative preamble part, respectively, with each being of *N* in length, as illustrated in Figure 1b. The calculation begins when the first nonzero entry of the preamble falls into the previous positive SAC window, and ends when the last nonzero entry of the preamble leaves the latter negative SAC window. Hence, with the total preamble length being 2N, the SAC operation during the synchronization of the Minn’s method requires $T_{\mathrm{mul}}^{\left(M\right)}=2.5N$ times of multiplications and $T_{\mathrm{add}}^{\left(M\right)}=10N$ times of additions. Thus the total computational complexity of Minn’s method is in the order of O(N).

For the proposed method, as described in Section 4.1, there are three SAC windows $R_{1,n}$, $R_{2,n}$, and $R_{3,n}$, with each being of length *N*. The calculation of SAC begins when the first nonzero entry of the preamble falls into the $R_{3,n}$ SAC window, and ends when the last nonzero entry of the preamble leaves the $R_{1,n}$ SAC window. Hence, with the total preamble length also being 2N, the SAC operation during the synchronization of the proposed method requires $T_{\mathrm{mul}}^{\left(P\right)}=9N$ times of multiplications and $T_{\mathrm{add}}^{\left(P\right)}=18N$ times of additions in total. Thus the total computational complexity of the proposed method is also in the order of O(N).

Although there is a moderate complexity increase to the proposed method due to more correlation and multiplication operations, both the proposed and conventional methods have a computational complexity in the order of O(N). Hence, the cost of calculation resource is in the same order, and the proposed method is applicable for practical system implementation.

## 5. Performance Evaluation

Extensive simulations are carried out to compare the performance of the conventional and the proposed methods in the presence of the NBI under different channel conditions. In this work, we conducted simulation tests that are consistent with the theoretical models and methods provided to evaluate the performance. As claimed in Section 2, note that all the methods are simulated with the same preamble length for fair comparison. The simulation setup is configured in a typical PLC transmission system, with the parameters listed in Table 1. A multipath PLC channel model defined in [26] is applied to evaluate the detection algorithm in multi-path and power line transmission environments, with the profile of parameters listed in Table 2. The length of the frequency–domain TS for the OFDM preamble is set as L=192, so at least the 7-bit signaling information can be conveyed. The simulations consider introducing an NBI with the power of −12 dB with respect to the average signal power, in order to evaluate the capability of different methods against the NBI.

**Figure 6:**
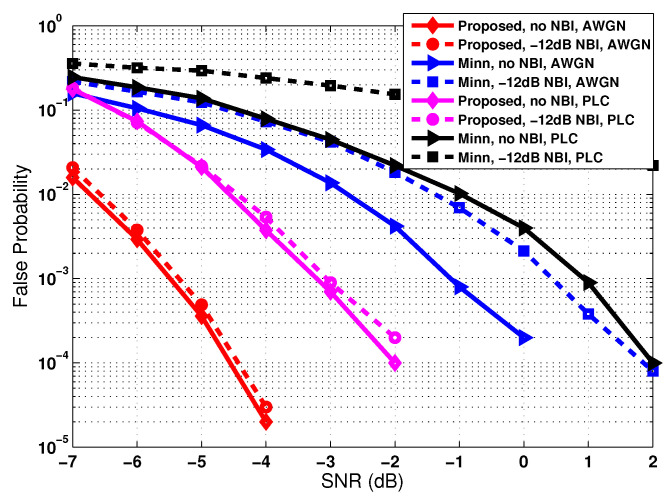
False probability of preamble detection in AWGN and PLC channels when the missed probability is around $10^{-3}$.

The false probability for the SAC peak detection is simulated to evaluate the detection performance of the proposed and conventional preambles. As shown in Figure 6, at the target missed probability of $10^{-3}$, the false probability of the proposed method is compared with that of Minn’s method. It can be observed from Figure 6 that the false probability of the proposed method has a 4 dB gain over Minn’s method under AWGN and PLC channels in the absence of the NBI. When the NBI is present, which is marked by dashed lines in Figure 6, it is shown that the proposed method is hardly affected by the NBI, whereas Minn’s method suffers from a degradation of more than 1 dB under AWGN channel and more than 4 dB under the PLC channel.

**Figure 7:**
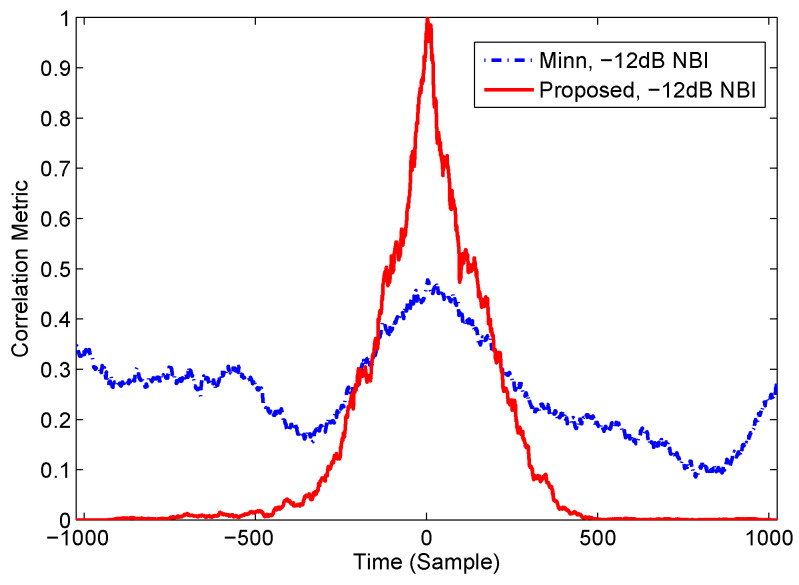
Timing correlation peaks for two methods in the presence of NBI.

In order to investigate the SAC peak performance of different preambles in the presence of the NBI, as depicted in Figure 7, the SAC peaks of Minn’s and the proposed methods in the presence of the −12 dB NBI are simulated, which is compared with the case without NBI in Figure 4. It can be noted from Figure 7 that the SAC peak of Minn’s method in the presence of NBI is significantly smaller than that without NBI in Figure 4. Moreover, the sub-peaks of Minn’s method become stronger and the main peak becomes less sharper with respect to that without NBI, making the false timing detection probability increase. On the other hand, the SAC peak of the proposed method is almost not affected by the −12 dB NBI, so the timing performance in the presence of the NBI is guaranteed.

**Figure 8:**
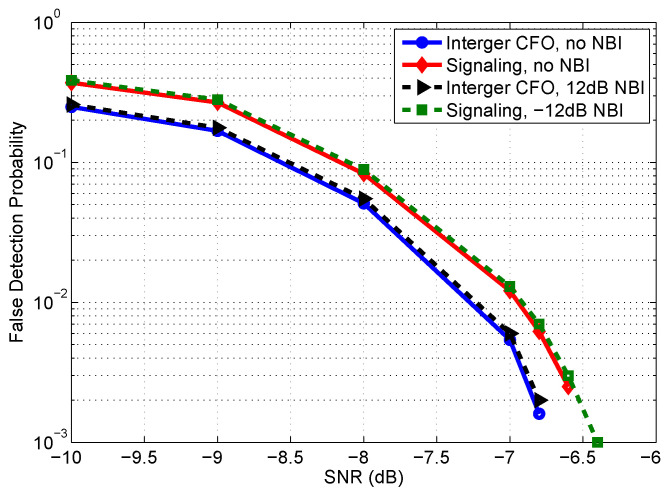
False probability of integer CFO estimation and signaling detection.

Finally, to investigate the performance of the integer CFO estimation and signaling detection, their false probabilities are simulated and presented in Figure 8. It is observed from Figure 5 and Figure 8 that, if the preamble is correctly detected, it is feasible to accurately estimate both the integer CFO and the signaling information. One should note that, the signaling can be decoded correctly if and only if both pairs of peaks shown in Figure 5 are detected correctly. Therefore, one can derive the approximate relation between the false probability of integer CFO estimation $P_{f,\mathrm{IntCFO}}$ and the false probability of signaling detection $P_{f,\mathrm{Sig}}$, as given by

(18) $$P_{f,\mathrm{Sig}}=1-{\left(1-P_{f,\mathrm{IntCFO}}\right)}^{2}.$$

From the simulation results in Figure 8, one can find that the simulation results are consistent with the theoretical analysis.

From the simulation results, we can see that the proposed scheme has superior performance in the accuracy of preamble detection in the presence of NBI compared with the benchmark scheme of Minn’s method. It should also be noted that, the performance gain of the proposed method is achieved at the cost of the increase in computational complexity. As mentioned in Section 4, the proposed timing scheme requires three pairs of cyclic parts in the calculation of the SACs, which is more than that of Minn’s method. The scrambling operation also brings extra complexity to the scheme. Hence, the proposed scheme is more suitable for the PLC system with a moderate computational capability but requires more accurate timing performance.

## 6. Conclusions

A novel preamble structure is proposed for OFDM-based PLC systems in the smart grid to improve the accuracy of timing and frequency synchronization in the presence of the NBI, and meanwhile capable of transmitting accurate signaling information immune to the NBI. The proposed preamble is shown through theoretical analysis and simulation results to enjoy significant better performance in timing and carrier frequency synchronization compared with conventional popular methods, with a moderate cost of computational complexity. Exploiting the proposed simple but crucial scrambling and desrambling operation, the proposed method is capable of combating against the NBI in various communication environments effectively. In addition, the proposed preamble simultaneously conveys signaling information by exploiting the frequency–domain designed sub-carrier pattern, which is robust to the NBI contamination as well. It is shown by the simulation results that the proposed method is hardly affected by the NBI, whereas Minn’s method suffers from a degradation of more than 1 dB under the AWGN channel and more than 4 dB under the PLC channel. Furthermore, the proposed preamble design can be applied to various communication systems suffering from the NBI. In future work following the method introduced in this paper, the proposed scheme can be tested on a real PLC system to evaluate the performance and obtain results in realistic PLC environments.

## Figures and Tables

**Table 1:** Simulation parameter.

Parameter	Value
NBI power	−12 dB
Symbol Rate	7.56 MSymbol/s
Bandwidth	8 MHz
Carrier Frequency	6 MHz
CFO	30 kHz
Symbol Duration	270.9 μs
Length of TS	192
FFT Size	1024

**Table 2:** PLC channel parameter profile.

Path Index	$d_{i}$ (m)	$g_{i}$		
1	200	0.64	*k*	1.0
2	222.4	0.38	$a_{0}$ (s/m)	0
3	244.8	−0.15	$a_{1}$ (s/m)	$7.8\times 10^{-10}$
4	267.5	0.05		

## Appendix A

The proof of the validity and capability of the proposed method against the NBI in multi-path channels is presented as follows.

First, we look at the SAC window $R_{1,n}$ at the desired timing position $n_{0}$ as given by (12). Taking the frequency-selective fading channel condition into consideration with the CIR denoted by ${\left\{h_{k}\right\}}_{k=0}^{L_{h}-1}$, the SAC result is derived as given by (A3), where the cross terms between the NBI and the preamble sequence can still be eliminated by sum averaging and de-scrambling as they can be in the AWGN channel shown by (12) because they are incoherent signals. The NBI component as pointed out above is also eliminated through de-scrambling and sum averaging as well. Hence, the SAC result of $R_{1,n}$ yields the peak value as given by (A3) at the desired timing position $n_{0}$, which is free from the impacts of the NBI.

Similarly, we have

(A1) $$R_{2,n}\approx -\sum_{l=0}^{N/2-1}\sum_{k=0}^{L_{h}-1}\sum_{m=0}^{L_{h}-1}{\left(-1\right)}^{k}h_{k}^{*}h_{m}x_{l-k}^{*}x_{l-m}$$

(A2) $$R_{3,n}\approx \sum_{l=0}^{N/2-1}\sum_{k=0}^{L_{h}-1}\sum_{m=0}^{L_{h}-1}{\left(-1\right)}^{k}h_{k}^{*}h_{m}x_{l-k}^{*}x_{l-m}$$

(A3) $$\begin{array}{c} R_{1,n_{0}}=\sum_{l=0}^{N/2-1}{\left(-1\right)}^{l}r_{n_{0}+l}^{*}\cdot r_{n_{0}+l-N/2}\\ =\sum_{l=0}^{N/2-1}{\left(-1\right)}^{l}{\left[\sum_{k=0}^{L_{h}-1}h_{k}\cdot {\left(-1\right)}^{l-k}x_{l-k}+I_{0}\exp\left(j2\pi f_{\mathrm{NB}}l\right)\right]}^{*}\cdot \\ \left[\sum_{m=0}^{L_{h}-1}h_{m}\cdot x_{l-m}+I_{0}\exp\left(j2\pi f_{\mathrm{NB}}\left(l-N/2\right)\right)\right]\\ =\sum_{l=0}^{N/2-1}\left[{\left(-1\right)}^{l}{\left(\sum_{k=0}^{L_{h}-1}h_{k}\cdot {\left(-1\right)}^{l-k}x_{l-k}\right)}^{*}\left(\sum_{m=0}^{L_{h}-1}h_{m}\cdot x_{l-m}\right)\right.\\ +{\left(-1\right)}^{l}{\left(\sum_{k=0}^{L_{h}-1}h_{k}\cdot {\left(-1\right)}^{l-k}x_{l-k}\right)}^{*}I_{0}\exp\left(j2\pi f_{\mathrm{NB}}\left(l-N/2\right)\right)\\ +{\left(-1\right)}^{l}\left(\sum_{m=0}^{L_{h}-1}h_{m}\cdot x_{l-m}\right)I_{0}\exp\left(-j2\pi f_{\mathrm{NB}}l\right)\left.+{\left(-1\right)}^{l}\underset{\mathrm{NBI}\;\mathrm{component}}{\underset{︸}{I_{0}^{2}\exp\left(-j2\pi f_{\mathrm{NB}}N/2\right)}}\right]\\ \approx \sum_{l=0}^{N/2-1}\sum_{k=0}^{L_{h}-1}\sum_{m=0}^{L_{h}-1}{\left(-1\right)}^{k}h_{k}^{*}h_{m}x_{l-k}^{*}x_{l-m}, \end{array}$$

(A4) $$\begin{array}{c} R_{c,n_{0}^{{}^{\prime }}}=\sum_{l=0}^{L_{c}-1}r_{n_{0}^{{}^{\prime }}+l}^{}\cdot r_{n_{0}^{{}^{\prime }}+l-N_{c}}^{*}\\ =\sum_{l=0}^{L_{c}-1}\left[\sum_{m=0}^{L_{h}-1}h_{m}\cdot x_{l-m}+I_{0}\exp\left(j2\pi f_{\mathrm{NB}}l\right)\right]\cdot {\left[\sum_{k=0}^{L_{h}-1}h_{k}\cdot x_{l-k}+I_{0}\exp\left(j2\pi f_{\mathrm{NB}}\left(l+N_{c}\right)\right)\right]}^{*}\\ =\sum_{l=0}^{L_{c}-1}\left[{\left(\sum_{k=0}^{L_{h}-1}h_{k}\cdot x_{l-k}\right)}^{*}\left(\sum_{m=0}^{L_{h}-1}h_{m}\cdot x_{l-m}\right)\right.+{\left(\sum_{k=0}^{L_{h}-1}h_{k}\cdot x_{l-k}\right)}^{*}I_{0}\exp\left(j2\pi f_{\mathrm{NB}}l\right)\\ \;\;\;\;\;\;+\left(\sum_{m=0}^{L_{h}-1}h_{m}\cdot x_{l-m}\right)I_{0}\exp\left(-j2\pi f_{\mathrm{NB}}\left(l+N_{c}\right)\right)\left.+\underset{\mathrm{NBI}\;\mathrm{component}}{\underset{︸}{I_{0}^{2}\exp\left(-j2\pi f_{\mathrm{NB}}N_{c}\right)}}\right]\\ \approx \sum_{l=0}^{N/2-1}\left(\sum_{k=0}^{L_{h}-1}\sum_{m=0}^{L_{h}-1}h_{k}^{*}h_{m}x_{l-k}^{*}x_{l-m}+\underset{\mathrm{NBI}\;\mathrm{component}}{\underset{︸}{I_{0}^{2}\exp\left(-j2\pi f_{\mathrm{NB}}N_{c}\right)}}\right). \end{array}$$

Therefore, all the three SAC results are free from the impacts of the NBI, making the final peak value $R_{3,{\hat{n}}_{0}}$ immune to the NBI, so the estimated timing position ${\hat{n}}_{0}$ accurately matches the desired timing position $n_{0}$ in the presence of the NBI under the multi-path channel.

The analysis for the contamination of the NBI upon the conventional methods as given by (3) can be extended to multi-path channels through similar reasoning. Considering multi-path fading channel, the SAC result $R_{c,n}$ of the conventional methods at the desired timing position $n_{0}^{{}^{\prime }}$ is given by (A4).

From (A4), it can be noted that, although the cross terms between the NBI and the preamble sequences are eliminated through sum averaging, the NBI component as pointed out above remains in the SAC result because there is no scrambling to eliminate it. Hence, under the multi-path fading channel, the remained NBI component will cause serious impacts on the peak value, so the conventional timing synchronization method will be seriously affected, which is similar to the result for the AWGN channel described by (3).
